# Mediterranean Style Diet and 12-Year Incidence of Cardiovascular Diseases: The EPIC-NL Cohort Study

**DOI:** 10.1371/journal.pone.0045458

**Published:** 2012-09-28

**Authors:** Marieke P. Hoevenaar-Blom, Astrid C. J. Nooyens, Daan Kromhout, Annemieke M. W. Spijkerman, Joline W. J. Beulens, Yvonne T. van der Schouw, Bas Bueno-de-Mesquita, W. M. Monique Verschuren

**Affiliations:** 1 Centre for Prevention and Health Services Research, National Institute for Public Health and the Environment, Bilthoven, The Netherlands; 2 Division of Human Nutrition, Wageningen University, Wageningen, The Netherlands; 3 Julius Center for Health Sciences and Primary Care, University Medical Center, Utrecht, The Netherlands; 4 Centre for Nutrition and Health, National Institute for Public Health and the Environment, Bilthoven, The Netherlands; 5 Department of Gastroenterology and Hepatology, University Medical Centre, Utrecht, The Netherlands; Brigham and Women’s Hospital and Harvard Medical School, United States of America

## Abstract

**Background:**

A recent meta-analysis showed that a Mediterranean style diet may protect against cardiovascular diseases (CVD). Studies on disease-specific associations are limited. We evaluated the Mediterranean Diet Score (MDS) in relation to incidence of total and specific CVDs.

**Methods:**

The EPIC-NL Study is a cohort of 40,011 men and women aged 20–70 years, examined between 1993 and 1997, with 10–15 years of follow-up. Diet was assessed with a validated food frequency questionnaire and the MDS was based on the daily intakes of vegetables, fruits, legumes and nuts, grains, fish, fatty acids, meat, dairy, and alcohol. Cardiovascular morbidity and mortality were ascertained through linkage with national registries. Cox proportional hazards models were used to estimate hazard ratios (HRs) and 95% confidence intervals (CI) adjusted for age, sex, cohort, smoking, physical activity, total energy intake, and educational level.

**Results:**

In 34,708 participants free of CVD at baseline, 4881 CVD events occurred, and 487 persons died from CVD. A two unit increment in MDS (range 0–9) was inversely associated with fatal CVD (HR: 0.78; 95%CI: 0.69–0.88), total CVD (HR: 0.95 (0.91–0.98)), myocardial infarction (HR: 0.86 (0.79–0.93)), stroke (HR: 0.88 (0.78–1.00)), and pulmonary embolism (HR: 0.74 (0.59–0.92)). The MDS was not related to incident angina pectoris, transient ischemic attack and peripheral arterial disease.

**Conclusion:**

Better adherence to a Mediterranean style diet was more strongly associated with fatal CVD than with total CVD. Disease specific associations were strongest for incident myocardial infarction, stroke and pulmonary embolism.

## Introduction

For a long time, the focus of nutritional research was on nutrients. However, interactions and correlations among nutrients will influence their bioavailability and absorption. [1], [2] Therefore, investigating the relation of single nutrients with cardiovascular diseases (CVD) is not sufficient, and also individual foods and food patterns need to be studied in relation to CVD incidence.

The traditional Mediterranean diet is a prototype of a healthy diet and is associated with a low risk of CVD. [3] Key features of a traditional Mediterranean diet are the use of olive oil as the principle component of fat; relatively high consumption of fruit, vegetables, fish, whole grains, legumes, and nuts; low consumption of meat and dairy; and moderate alcohol consumption with meals [4], [5].

Controlled trials showed that a Mediterranean style diet was associated with favorable changes in body weight, body mass index, systolic and diastolic blood pressure, fasting plasma glucose, total cholesterol and high-sensitivity C-reactive protein, which may all affect CVD incidence [6]. In a secondary prevention trial in myocardial infarction patients, a Mediterranean style diet enriched with alpha-linolenic acid was associated with a 73% lower risk of cardiac death and non-fatal myocardial infarction compared with a prudent diet [7], [8]. Also, in a cohort study on the Mediterranean diet and survival among Greek cardiac patients, a higher adherence to the Mediterranean diet was strongly inversely associated with cardiac death [9].

A recent meta-analysis showed that each two unit increment in the MDS was associated with a 10% lower incidence of CVD. [10] The meta-analysis combined studies with different fatal and nonfatal CVD enpoints. [10] The recently published prospective cohort study by Gardener et al. observed an association of the MDS with fatal CVD, but not with incident myocardial infarction or ischemic stroke. [11] These results suggest that a Mediterranean style diet may affect the risk of specific CVD differently. Gardener et al. concluded that studies on Mediterranean style diets in relation to specific CVD endpoints are needed. [11] Associations of Mediterranean style diets with angina pectoris, transient ischemic attack, peripheral arterial disease, or pulmonary embolism have not yet been investigated.

The purpose of the present study was to investigate a Mediterranean style diet, as a prototype of a healthy diet, in association with incident fatal CVD, total CVD, and with specific CVD. The secondary aim was to explore which of the components of the MDS contributed most to these associations by assessing the effect of alternately excluding components of the MDS.

## Methods

### Study Population

The EPIC-NL cohort is the Dutch contribution to the European Prospective Investigation into Cancer and Nutrition (EPIC) and consists of the Monitoring Project on Risk Factors for Chronic Diseases (MORGEN) and the PROSPECT cohort. Baseline data of the 40,011 participants were collected between 1993 and 1997. The MORGEN cohort consists of 22,654 men and women aged 20–65 years who were recruited through random population sampling in three Dutch towns (Amsterdam, Maastricht and Doetinchem). [12] The PROSPECT cohort included 17,357 women aged 50–70 years, who participated in a breast cancer screening program in the province of Utrecht.

We excluded participants with prevalent CVD or type 2 diabetes based on self-report and hospital admission data (n = 1401), women who were pregnant at baseline (n = 140), those with extremely low or high reported energy intakes (ie, those in the lowest and highest 0.5% of the ratio of energy intake over basal metabolic rate) (n = 373), those with no information on dietary intake or any of the covariates (n = 855), as well as those with no information on vital status or cardiovascular events (n = 2,534). In total 34,708 participants remained for the analyses.

### Ethics Statement

All participants signed an informed consent form prior to study inclusion. The study complies with the Declaration of Helsinki and was approved by two medical ethics committees [12].

### Mediterranean Style Diet

Dietary intake was assessed by a validated [13], [14] food frequency questionnaire (FFQ) which contained questions on the habitual frequency of consumption of 178 food items during the year preceding enrollment. Additional information was obtained on consumption frequency for different sub-items, and preparation methods. Colored photographs were used to estimate portion sizes of 28 food items. [13], [14] Reproducibility and validity were assessed in 121 men and women. [13], [14] Median twelve-month reproducibility of 16 food groups was 0.71 for men and 0.77 for women, ranging from resp. 0.45 and 0.63 for fish to 0.83 and 0.92 for alcoholic beverages. [13] The median validity of these 16 food groups, with 12 monthly 24-hour recalls as reference, was 0.61 for men and 0.53 for women, ranging from 0.32 for fish in men and 0.31 for vegetables in women to 0.74 (men) and 0.87 (women) for alcoholic beverages [13].

We operationalized the concept of a healthy diet with the modified Mediterranean Diet Score (MDS) defined by Trichopoulou et al. [5] For the composition of this score, values of 0 or 1 were assigned to each nutritional component using the sex-specific medians as cut-off values. For vegetables, fruits, legumes and nuts combined, grains, fish and seafood combined, and the ratio of unsaturated to saturated fatty acids, intakes equal to or above the median were assigned a value of 1, and for intakes below that median a value of 0. For meat and for dairy products the scoring was inverted. We dichotomized alcohol consumption into non-users and users, because of the relatively low levels of alcohol consumption in this population. We did not define an upper limit for alcohol consumption, due to the low levels of alcohol consumption in the present population, and called the alcohol consumption of our population moderate. A value of 1 was assigned for consuming at least one drink per month and a value of 0 for consuming less than 1 drink per month. The modified MDS could take a value from 0 (minimal adherence) to 9 (maximal adherence) [5] and associations with CVD were assessed in categories 0–2, 3–4, 5–6 and 7–9 and by two unit increment.

### Ascertainment of Fatal and Nonfatal CVD Events

Participants were followed to the first nonfatal cardiovascular event, death, emigration, or were censored at January 1^st^ 2008. Morbidity data were provided by the National Medical Registry (NMR) using the Dutch Hospital Discharge data. Eighty-eight percent of the hospital admissions could uniquely be linked to an individual on the basis of sex, date of birth, and postal code. [15] In a validation study conducted in a subsample of this population, the Hospital Discharge data was compared with that of a detailed clinical registry. [16] A sensitivity of 84% was observed for acute myocardial infarction hospital admissions. [16] We obtained vital status through linkage with municipal population registries. Subsequently, primary (underlying) and secondary causes of death were obtained through linkage with data from ‘Statistics Netherlands’. [12] In a study in which causes of death were coded again two years after initial coding, agreement ranged from 77–89% for CVD [17].

Incidence was defined as both fatal and nonfatal events unless otherwise specified. For fatal and nonfatal incidence combined, only first events were taken into account. For instance, when a person experienced a myocardial infarction followed by a pulmonary embolism, this person was censored after the myocardial infarction. For fatal CVD, no exclusions were made due to previous nonfatal events during follow-up. Total CVD was coded as ICD9 [18] codes 390–459 and 798. Myocardial infarction was coded as 410–412 and 414, angina pectoris as 413, stroke as 430–434, and 436, transient ischemic attack as 435, peripheral arterial disease as 440–444, and pulmonary embolism as 415.1. The remaining CVD codes were coded as other CVD. Composite CVD consisted of fatal CVD, plus nonfatal first myocardial infarction and stroke. Causes of death after 1996 were coded according to the corresponding ICD10 [19] codes.

### Covariates

A self-administered general questionnaire provided information on educational level, smoking status, and physical activity. [12] Educational level was operationalized as low (lower vocational training or primary school), medium (secondary school and intermediate vocational training), or high (higher vocational training or university) and cigarette, cigar or pipe smoking as current, former or never. Physical activity was assessed using the validated [20] EPIC questionnaire and dichotomized according to the Cambridge Physical Activity Index (CPAI) into (moderately) active and (moderately) inactive. [21] In the first year of the MORGEN study (1993, 14.2% of the study population), physical activity was not assessed with the EPIC questionnaire. The missing physical activity data were imputed using single imputation as previously described (SPSS MVA procedure) [22].

### Statistical Analysis

Statistical analyses were performed using SAS 9.2 software (SAS Institute, INC., Cary, NC). Participants’ characteristics were calculated by sex and cohort as means (standard deviation) or medians (inter-quartile range) for continuous variables, and as percentages for categorical variables. Cox proportional hazards models were used to estimate hazard ratios (HRs) and 95% confidence intervals (CI). The proportional hazard assumption was fulfilled according to the graphical approach and according to Schoenfeld residuals. Two consecutive models were used to assess the associations between the MDS and the CVD endpoints. The first model was adjusted for age, sex and cohort. The second model was additionally adjusted for smoking, physical activity, total energy intake, and educational level. We did not include BMI, blood pressure or serum cholesterol in our analyses, since we consider these intermediates in the association between a Mediterranean style diet and CVD. By adding interaction terms to the models, we assessed interaction on a multiplicative scale for age, sex, and cohort in the association between the MDS and the different cardiovascular endpoints. We also explored which of the components of the MDS contributed most to the associations for those CVD that were related to the MDS at p<0.10. For this, we alternately excluded one component of the MDS while adjusting for the excluded component. This reduced the ten point score (0–9) to a nine point score (0–8). To preserve comparability between the ten point and the nine point scores, we multiplied the logarithm of the estimated nine mortality ratios by 9/10 before exponentiating them [23].

To minimize the possibility that dietary habits had changed in response to development of intermediate symptoms (e.g. hypertension) during follow-up (reversed causation), the analyses were repeated after exclusion of persons with an event in the first two years of follow-up.

## Results

During 10–15 years of follow-up (mean 11.8 years) 4881 CVD events occurred, and 487 persons died from CVD. **Table 1** shows the distribution of participants’ characteristics and dietary intake stratified by sex and cohort. 25% of the population was male. Mean age at baseline was 43 years for men, 42 years for women in MORGEN and 58 years for women in PROSPECT. Dietary intake was similar in women in MORGEN and PROSPECT, with the exception of fruit consumption, which was higher in PROSPECT.

**Table 1:** Participants’ characteristics at baseline and CVD incidence, by sex and cohort, the EPIC-NL Study.

	Men (MORGEN)	Women (MORGEN)	Women (PROSPECT)
**N**	8764	10537	15407
**Age (years)** a	43 (11)	42 (11)	58 (6)
**Education (% low)** b	32	35	45
**Smoking (%)** c	38	35	23
**Physically active (%)**	71	68	67
**Alcohol consumption (%, ≥1 glass per month)**	91	77	78
**Alcohol (gram/day)** a	12 (3–25)	3 (0–11)	4 (1–13)
**Energy intake (kcal/day)** a	2518 (2139–2987)	1932 (1635–2265)	1763 (1508–2046)
**Vegetables (gram/day)** a	103 (77–134)	113 (86–147)	123 (95–158)
**Fruit** **(gram/day)** a	117 (51–186)	124 (78–239)	224 (119–295)
**Legumes** **(gram/day)** a	15 (8–24)	12 (6–20)	12 (6–20)
**Nuts** **(gram/day)** a	7 (2–16)	4 (2–10)	4 (1–8)
**Grains** **(gram/day)** a	250 (188–319)	184 (142–234)	147 (115–184)
**Fish and seafood** **(gram/day)** a	8 (3–14)	7 (3–14)	8 (3–16)
**Unsaturated fatty acids** **(gram/day)** a	58 (47–71)	44 (36–54)	36 (29–44)
**Saturated fatty acids** **(gram/day)** a	41 (33–50)	32 (25–39)	28 (22–34)
**Dairy and dairy products** **(gram/day)** a	353 (196–578)	346 (196–548)	414 (267–597)
**Meat products** **(gram/day)** a	141 (105–179)	102 (63–131)	87 (54–118)
**Fatal CVD (n/%)** d	136/1.6	70/0.7	281/1.8
**Incident CVD (n/%)** d	1202/13.7	1078/10.2	2601/16.9
**Composite CVD (n/%)** d,e	548/6.3	317/3.0	900/5.8
**Incident MI (n/%)** d	390/4.5	184/1.8	496/3.2
**Incident AP (n/%)** d	116/1.3	70/0.7	193/1.3
**Incident stroke (n/%)** d	93/1.1	95/0.9	260/1.7
**Incident TIA (n/%)** d	32/0.4	32/0.3	90/0.6
**Incident PAD (n/%)** d	102/1.2	48/0.5	135/0.9
**Incident PE (n/%)** d	25/0.3	35/0.3	71/0.5
**Incident other CVD (n/%)** d	450/5.1	621/5.9	1376/8.9

a Numbers are given as mean (sd) or as median (interquartile range);

b Low educational level: lower vocational training or primary school;

c Cigarette, cigar or pipe;

d See method section for ICD codes;

e Composite of fatal CVD, nonfatal myocardial infarction and nonfatal stroke.

CVD = cardiovascular diseases, MI = myocardial infarction, AP = angina pectoris, TIA = transient ischemic attack, PAD = peripheral arterial disease, PE = pulmonary embolism.

After adjustment for all confounders, each two unit increment in MDS was associated with a 22% lower incidence of fatal CVD (HR: 0.78; 95%CI: 0.69–0.88), a 5% lower incidence of total CVD (HR: 0.95 (0.91–0.98)), and a 15% lower incidence of composite CVD (HR: 0.85 (0.80–0.91)) (**Table 2**). For specific CVD, statistically significant associations were observed for incident myocardial infarction (HR: 0.86 (0.79–0.93)), stroke (HR: 0.88 (0.78–1.00)), and pulmonary embolism (HR: 0.74 (0.59–0.92)). The strength of the association of the MDS with ischemic (HR: 0.86 (0.72–1.01)) did not differ from that with hemorrhagic stroke (0.87 (0.60–1.09)). The association with pulmonary embolism was not linear: the largest decrease in HR was between the MDS categories ‘0–2’ and ‘3–4’. Also, the association with pulmonary embolism was stronger for men (HR: 0.39 (0.23–0.67) per two unit increment) than for women (HR 0.84 (0.65–1.07)) (p for interaction: <0.01). Adherence to a Mediterranean style diet was not related to incident angina pectoris, transient ischemic attack and peripheral arterial disease. We observed no other interactions with age, sex (except for pulmonary embolism) or cohort. Exclusion of cases in the first two years of follow-up hardly changed our results (data not shown).

**Table 2:** Hazard ratios (95%CI) of specific CVD by MDS category and by two unit increment in MDS, The EPIC-NL Study.

	MDS				
	0–2	3–4	5–6	7–9	By two unit increment
Persons at risk, no.	2469	12249	14832	5158	34708
Person years total CVD	28026	139539	170208	58924	396697
**Fatal CVD, no.**	62	206	178	41	487
Model 1^a^	1.00 (ref)	0.67 (0.50–0.89)	0.51 (0.38–0.69)	0.36 (0.24–0.54)	0.72 (0.64–0.81)
Model 2^b^	1.00 (ref)	0.72 (0.54–0.96)	0.60 (0.44–0.80)	0.44 (0.30–0.66)	0.78 (0.69–0.88)
**Incident CVD, no.**	438	1820	1973	650	4881
Model 1^a^	1.00 (ref)	0.84 (0.75–0.93)	0.77 (0.69–0.85)	0.75 (0.66–0.84)	0.90 (0.87–0.94)
Model 2^b^	1.00 (ref)	0.87 (0.79–0.97)	0.84 (0.75–0.93)	0.84 (0.75–0.96)	0.95 (0.91–0.98)
**Composite CVD** c **, no.**	186	699	684	196	1765
Model 1^a^	1.00 (ref)	0.75 (0.64–0.88)	0.63 (0.53–0.74)	0.54 (0.44–0.66)	0.80 (0.75–0.84)
Model 2^b^	1.00 (ref)	0.80 (0.68–0.94)	0.72 (0.61–0.85)	0.65 (0.53–0.80)	0.85 (0.80–0.91)
**Incident MI, no.**	106	428	412	124	1070
Model 1^a^	1.00 (ref)	0.79 (0.64–0.98)	0.64 (0.52–0.79)	0.57 (0.44–0.74)	0.79 (0.74–0.86)
Model 2^b^	1.00 (ref)	0.85 (0.69–1.05)	0.74 (0.60–0.92)	0.70 (0.54–0.92)	0.86 (0.79–0.93)
**Incident AP, no.**	20	138	171	50	379
Model 1^a^	1.00 (ref)	1.37 (0.86–2.20)	1.44 (0.91–2.29)	1.25 (0.74–2.10)	1.02 (0.90–1.17)
Model 2^b^	1.00 (ref)	1.44 (0.90–2.31)	1.59 (1.00–2.54)	1.44 (0.85–2.43)	1.08 (0.95–1.23)
**Incident stroke, no.**	46	174	177	51	448
Model 1^a^	1.00 (ref)	0.77 (0.55–1.06)	0.68 (0.49–0.94)	0.59 (0.39–0.87)	0.83 (0.74–0.93)
Model 2^b^	1.00 (ref)	0.82 (0.59–1.13)	0.77 (0.55–1.07)	0.70 (0.47–1.05)	0.88 (0.78–1.00)
**Incident TIA, no.**	13	59	55	27	154
Model 1^a^	1.00 (ref)	0.92 (0.51–1.68)	0.75 (0.41–1.37)	1.10 (0.57–2.13)	0.98 (0.80–1.20)
Model 2^b^	1.00 (ref)	0.94 (0.51–1.71)	0.77 (0.42–1.43)	1.15 (0.58–2.25)	1.00 (0.81–1.23)
**Incident PAD, no.**	32	111	109	33	285
Model 1^a^	1.00 (ref)	0.68 (0.46–1.01)	0.56 (0.38–0.84)	0.51 (0.31–0.83)	0.79 (0.68–0.92)
Model 2^b^	1.00 (ref)	0.77 (0.52–1.14)	0.73 (0.49–1.09)	0.74 (0.45–1.21)	0.91 (0.78–1.06)
**Incident PE, no.**	19	46	52	14	131
Model 1^a^	1.00 (ref)	0.49 (0.29–0.84)	0.47 (0.28–0.79)	0.37 (0.18–0.73)	0.77 (0.62–0.95)
Model 2^b^	1.00 (ref)	0.47 (0.28–0.81)	0.43 (0.25–0.74)	0.33 (0.16–0.67)	0.74 (0.59–0.92)
**Incident other CVD, no.**	206	873	1011	357	2447
Model 1^a^	1.00 (ref)	0.86 (0.74–1.01)	0.85 (0.73–0.99)	0.88 (0.74–1.04)	0.97 (0.93–1.02)
Model 2^b^	1.00 (ref)	0.88 (0.76–1.03)	0.88 (0.76–1.03)	0.93 (0.78–1.11)	0.99 (0.94–1.05)

a Model 1: analyses adjusted for age, sex and cohort;

b Model 2: model 1+ smoking, physical activity, energy intake and educational level.

c Composite of fatal CVD, nonfatal myocardial infarction and nonfatal stroke.

CVD = cardiovascular diseases, MI = myocardial infarction, AP = angina pectoris, TIA = transient ischemic attack, PAD = peripheral arterial disease, PE = pulmonary embolism.

In general, alternately excluding one of the components of the MDS did not materially change our results (**Table 3**). The exclusion of alcohol from the MDS had the largest impact on the associations of MDS with CVD, in particular for incident fatal CVD, total CVD, composite CVD and myocardial infarction. Excluding fish and seafood or the fatty acid ratio attenuated the association for pulmonary embolism most.

**Table 3:** Hazard ratios (95%CI) associated with two unit increment in MDS and after alternate exclusion of each of its components.a,b

MDS	Fatal CVD	Incident CVD	Composite CVD	Incident MI	Incident stroke	Incident PE
total	0.78 (0.69–0.88)	0.95 (0.91–0.98)	0.85 (0.80–0.91)	0.86 (0.79–0.93)	0.88 (0.78–1.00)	0.74 (0.59–0.92)
minus vegetables	0.79 (0.70–0.88)	0.94 (0.90–0.97)	0.86 (0.81–0.92)	0.86 (0.80–0.93)	0.90 (0.80–1.02)	0.71 (0.57–0.89)
minus fruits	0.78 (0.70–0.87)	0.95 (0.91–0.98)	0.87 (0.82–0.93)	0.88 (0.82–0.95)	0.91 (0.81–1.03)	0.76 (0.62–0.95)
minus legumes and nuts	0.77 (0.69–0.87)	0.94 (0.91–0.97)	0.83 (0.78–0.89)	0.84 (0.78–0.91)	0.86 (0.76–0.97)	0.73 (0.59–0.92)
minus grains	0.81 (0.72–0.90)	0.96 (0.93–1.00)	0.87 (0.82–0.92)	0.87 (0.81–0.94)	0.91 (0.81–1.02)	0.79 (0.64–0.98)
minus fish and seafood	0.81 (0.72–0.91)	0.95 (0.92–0.98)	0.87 (0.82–0.92)	0.86 (0.79–0.93)	0.92 (0.82–1.04)	0.81 (0.65–1.00)
minus fatty acid ratio	0.81 (0.72–0.92)	0.94 (0.90–0.98)	0.84 (0.79–0.89)	0.84 (0.77–0.91)	0.85 (0.75–0.96)	0.82 (0.65–1.03)
minus dairy products	0.79 (0.70–0.88)	0.94 (0.91–0.98)	0.85 (0.80–0.90)	0.86 (0.79–0.92)	0.85 (0.76–0.95)	0.75 (0.61–0.93)
minus meat products	0.79 (0.71–0.89)	0.96 (0.93–1.00)	0.88 (0.83–0.93)	0.89 (0.83–0.96)	0.91 (0.82–1.02)	0.73 (0.59–0.90)
minus alcohol	0.82 (0.74–0.92)	0.97 (0.93–1.00)	0.89 (0.84–0.94)	0.91 (0.84–0.98)	0.90 (0.80–1.01)	0.75 (0.61–0.93)

a Originally estimated logarithms of hazard ratios were multiplied by 9/10 and then exponentiated to correct for nine point scale.

b Hazard ratio (95% CI) adjusted for age, sex, cohort, smoking, physical activity, energy intake, educational level, and excluded component.

CVD = cardiovascular diseases, MI = myocardial infarction, PE = pulmonary embolism.

## Discussion

This study showed that a Mediterranean style diet was inversely associated with total CVD and more strongly so with fatal CVD. Inverse associations were also observed for composite CVD, myocardial infarction, stroke and pulmonary embolism. The MDS was not related to incident angina pectoris, transient ischemic attack and peripheral arterial disease. Alternate exclusion of components of the MDS showed that alcohol contributed most to the inverse association between MDS and CVD.

In the present study, a MDS of 7–9 was associated with a 56% lower incidence of fatal CVD compared to a MDS of 0–2, with a 16% lower incidence of total CVD and a 35% lower incidence of composite CVD. Comparison of our results with those of other investigations is hampered by differences in definition of Mediterranean style diet and of CVD endpoints. In previous studies, the MDS varied in definition of the components. Also, adherence to a Mediterranean style diet was categorized in various ways, ranging from two, to five categories of adherence. [10] The definition of CVD also varied among studies, with various combinations of ICD codes and inclusion of fatal or nonfatal CVD events. Taken together, results of previous studies showed that high compared to low adherence to a Mediterranean style diet was associated with a 20 [24] to 40% [25], [26] lower incidence of fatal CVD and a 20 [25] to 25% [11] lower incidence of composite CVD. These results are in line with those in the present study.

The association of the MDS with fatal CVD was stronger than with total CVD. A stronger association with fatal CVD may be due to the probabilistic linkage of the non-fatal Hospital Discharge data causing more misclassification than for fatal events. This may have resulted in weaker associations for nonfatal CVD. Furthermore, our results showed that adding ‘softer’ endpoints such as transient ischemic attack, angina pectoris and peripheral arterial disease to the composite of ‘hard’ endpoints reduced the strength of the associations considerably. Therefore, in discussing the strength of associations with different CVD endpoints, the definition of the latter is of utmost importance.

For the highest compared to the lowest MDS category we observed a 30% lower incidence of myocardial infarction and for each two unit increment a 14% lower incidence. This was in line with a recent cohort study in 2568 men and women in the United States by Gardener et al. who observed a 39% lower incidence of myocardial infarction for the highest compared to the lowest MDS category, and for each 1 unit increment a 6% lower incidence, though these associations were non-significant due to the small sample size. [11] Our results are also consistent with those observed in other cohort studies, in which high compared to low adherence to a Mediterranean style diet was associated with a 30–40% lower incidence of coronary heart disease [25], [26], [27], [28], [29], although coronary hart disease incidence was defined differently in the various studies.

The inverse association between the MDS and stroke incidence in the present study (highest compared to lowest category HR: 0.70 (0.47–1.05) and for a two unit increment HR: 0.88 (0.78–1.00)) is in agreement with the results Fung et al. obtained in the Nurses Health Study [25]. They observed a 13% lower incidence of stroke for those in the highest compared to the lowest quintile of adherence to a Mediterranean style diet. [25] The results of the cohort studies published so far, including ours (results not shown), showed similar results for ischemic and hemorrhagic stroke [11], [25].

We observed that the MDS was inversely associated with the incidence of pulmonary embolism. This association was stronger for men than for women. Diet is hypothesized to affect venous thromboembolism, and thereby pulmonary embolism, by altering levels of haemostatic and fibrinolytic factors. [30] To our knowledge other studies did not investigate diet in relation to pulmonary embolism before. However, we could compare our results with those of three large cohort studies investigating the relation of diet with venous thromboembolism. The results of these studies were inconsistent. [31], [32], [33] Therefore, our results for pulmonary embolism need confirmation by other prospective cohort studies.

In the present study, the MDS was not statistically significantly related to incident angina pectoris, transient ischemic attack and peripheral arterial disease. To our knowledge these associations have not been investigated earlier. These three diseases are ‘softer’ endpoints than e.g. myocardial infarction and stroke. This may have resulted in more misclassification [16] which may have diluted the associations of the MDS with these endpoints.

The associations of the MDS with fatal CVD, incident CVD, composite CVD, and myocardial infarction attenuated most when excluding alcohol from the MDS. Excluding fish and seafood or the fatty acid ratio attenuated the association with pulmonary embolism most. No previous study on the MDS in relation to CVD assessed the effect of alternately excluding components of the MDS. Trichopoulou et al. observed for the association between MDS and all-cause mortality also most attenuation after excluding alcohol consumption from the MDS. [23] Previous studies on a Mediterranean style diet assessing the contribution of its individual components to CVD incidence showed inconsistent results with respect to which component was strongest associated to CVD [11], [27], [29].

We studied the adherence to a Mediterranean style diet in a Dutch population. The Dutch diet is characterized by a low consumption of plant foods and fish and by a high consumption of animal foods compared to the traditional Mediterranean diet. [34] However, similar associations of a Mediterranean style diet with CVD were observed in Mediterranean, Northern European and in American populations. [10] This implies that, at different levels of adherence, a Mediterranean style diet is beneficial in relation to cardiovascular health. Furthermore, our associations were robust since in sensitivity analyses, including only whole grain cereals in the component ‘cereals’, or only moderate to high fat meat products and dairy products in the components ‘meat products’ and ‘dairy products’, hardly changed the results (results not shown). Also additionally adjusting the components of the MDS for energy intake (density method) or exclusion of cases in the first two years of follow-up hardly changed our results (results not shown).

Some limitations of our study need to be addressed. Dietary intake was self-reported using a validated food-frequency questionnaire (FFQ). This questionnaire had a good reproducibility, although the validity of vegetable (Spearman correlation coefficients: 0.38 for men and 0.31 for women) and fish (0.32 for men and 0.37 for women) consumption is of concern. [13], [14] In addition, diet was assessed only once and may have changed during follow-up, resulting in non-differential misclassification that may have attenuated the observed associations. Furthermore, our study is a prospective cohort study in which adherence to MDS was not randomized. Therefore, residual confounding cannot be ruled out. With regard to the cardiocascular follow-up, ‘hard’ endpoints, like myocardial infarction or stroke, are easier to diagnose than ‘softer’ endpoints like angina pectoris, transient ischemic attack or peripheral arterial disease. Also, ‘hard’ endpoints are more likely to be treated in the hospital than ‘softer’ ones, and thus monitored in the Hospital Discharge Registries [16]. In a validation study comparing the Hospital Discharge data to that of a cardiology information system, sensitivity was considerably larger for acute myocardial infarction (84%) than for unstable angina pectoris (53%) [16]. Therefore, misclassification is likely smaller for the ‘hard’ events, which may have resulted in stronger associations.

The present study also has advantages. EPIC-NL is a large prospective cohort study, especially designed to study associations between diet and chronic diseases, and included both men and women from the general population, with a broad age range and a long follow-up period. Because of the detailed cardiovascular follow-up data and the large sample size, we were able to investigate associations of a Mediterranean style diet with specific CVD.

In conclusion, the present study showed that better adherence to a Mediterranean style diet was more strongly associated with fatal CVD than with total CVD. Disease specific associations were strongest for incident myocardial infarction, stroke and pulmonary embolism.
